# The Data-Adaptive Fellegi-Sunter Model for Probabilistic Record Linkage: Algorithm Development and Validation for Incorporating Missing Data and Field Selection

**DOI:** 10.2196/33775

**Published:** 2022-09-29

**Authors:** Xiaochun Li, Huiping Xu, Shaun Grannis

**Affiliations:** 1 Department of Biostatistics and Health Data Science Indiana University School of Medicine The Richard M. Fairbanks School of Public Health Indianapolis, IN United States; 2 Data and Analytics Regenstrief Institute Inc. Indiana University School of Medicine Indianapolis, IN United States

**Keywords:** record linkage, Fellegi-Sunter model, latent class model, missing at random, matching field selection

## Abstract

**Background:**

Quality patient care requires comprehensive health care data from a broad set of sources. However, missing data in medical records and matching field selection are 2 real-world challenges in patient-record linkage.

**Objective:**

In this study, we aimed to evaluate the extent to which incorporating the missing at random (MAR)–assumption in the Fellegi-Sunter model and using data-driven selected fields improve patient-matching accuracy using real-world use cases.

**Methods:**

We adapted the Fellegi-Sunter model to accommodate missing data using the MAR assumption and compared the adaptation to the common strategy of treating missing values as disagreement with matching fields specified by experts or selected by data-driven methods. We used 4 use cases, each containing a random sample of record pairs with match statuses ascertained by manual reviews. Use cases included health information exchange (HIE) record deduplication, linkage of public health registry records to HIE, linkage of Social Security Death Master File records to HIE, and deduplication of newborn screening records, which represent real-world clinical and public health scenarios. Matching performance was evaluated using the sensitivity, specificity, positive predictive value, negative predictive value, and F1-score.

**Results:**

Incorporating the MAR assumption in the Fellegi-Sunter model maintained or improved F1-scores, regardless of whether matching fields were expert-specified or selected by data-driven methods. Combining the MAR assumption and data-driven fields optimized the F1-scores in the 4 use cases.

**Conclusions:**

MAR is a reasonable assumption in real-world record linkage applications: it maintains or improves F1-scores regardless of whether matching fields are expert-specified or data-driven. Data-driven selection of fields coupled with MAR achieves the best overall performance, which can be especially useful in privacy-preserving record linkage.

## Introduction

Quality patient care requires comprehensive health care data from a broad set of sources. Electronic medical record (EMR) data are increasingly distributed across many sources as the era of digital health care is accelerated in the United States. However, EMR data from independent databases often lack a common patient identifier, which impedes data aggregation, causes inefficiencies (eg, tests repeated unnecessarily), affects patient care, and hinders research. Record linkage is a requisite step for effective and efficient patient care and research. Without a unique universal patient identifier, linkage of patient records is a nontrivial task. The simplest class of approaches is the deterministic method, which requires the strict identity of the selected data elements of a pair of records, such as name, birthdate, gender, and Social Security number. Although deterministic algorithms are generally simple to implement and achieve excellent specificity, they have low sensitivity, are not robust to missing data, cannot quantify the uncertainty of the matching process, and are inflexible to changing data characteristics.

The Fellegi-Sunter (FS) [1] model is widely used for probabilistic record linkage based on the binary agreement or disagreement of a select set of fields of record pairs, such as Social Security number, first name, middle name, last name, and date of birth. The FS model is in essence a latent class model applied to record linkage problems. The latent class variable is the unobserved true match status, and the parameters in the model are the match prevalence, probabilities of field agreements among true matches (m-probabilities), and probabilities of field agreements among nonmatches (u-probabilities). A record pair’s matching weights are defined as the logarithms of the m- and u-probability ratios, and the sum of the weights is the matching score of the pair. Record pairs were then classified into matches and nonmatches based on their matching scores for a given threshold. The linking algorithm based on the FS model is shown to outperform the deterministic algorithm [2]. However, methodological gaps exist in configuring and applying the FS model.

First, it is well known that missing data are prevalent in real-world data in EMRs [3]. Data necessary for matching records are often missing from clinical data for many reasons: values may be coded as “unknown,” nonexistent (a person with no middle name), or omitted due to privacy concerns (such as Social Security number). Missing field values decrease the information content in the data and consequently hinder matching accuracy. Matching only records with full information is undesirable because it excludes many records and thus misses matches. One study found that mother’s date of birth was often absent because it was not the focus of pediatricians’ attention [4]. However, this information significantly improved the linkage procedure when present. Therefore, effective accommodation of missing data is needed to maximize linkage. Common strategies in practice involve excluding records with missing values in any of the matching fields when estimating match weights [5] or considering the missing field’s agreement pattern as disagreement [6] (missing as disagreement [MAD]). The former lacks efficiency because of the loss in sample size due to exclusion. The latter does not account for the fact that true matches can contain missing fields and is deficient in a theoretical justification. Another strategy is to model missing data in a matching field as the third category, in addition to the categories of agree and disagree [7]. However, it is well established that including missing data by adding a category “missing” causes serious biases, even when data are missing completely at random [8-13]. In a model-based approach, Enamordo et al [14] assume that data in matching fields are missing at random (MAR) conditional on the true match status. Their comprehensive simulation studies show that the FS model with MAR incorporated outperforms deterministic linkage in social science when linking voter files. How the FS model with MAR incorporated compares with the FS model using zero-filled data in which missing values in the original data are replaced by 0 by MAD has not been evaluated. Furthermore, while MAR is evaluated and applied to voting registries, its performance in linking EMR files is not known.

Second, although there may be numerous fields (or attributes) across record files not all of them are useful for matching. For example, if matching 2 obstetrics and gynecology databases, the field “gender” is not informative. In real-world data, there are likely also dependencies among the data fields. As we have demonstrated [15], the FS model exhibits poor matching accuracy when the fields are highly correlated. As more fields are used in the FS model, more dependencies may be introduced. Ideally, the FS model should be able to use a minimally sufficient set of fields. However, we are unaware of data-driven methods for matching field selection. In practice, the expert input is solicited to identify an appropriate subset matching fields. Several iterations may be required to achieve the desired match accuracy using a manually reviewed data set with known match statuses among record pairs. This process is neither scalable nor generalizable and is infeasible in privacy-preserving record linkage [7]. We are also unaware of any work that evaluates the effects of missing data treatment and field selection for matching simultaneously.

We will evaluate the effects of incorporating missing data treatment and matching field selection into the FS algorithm on linkage performance using 4 real-world use cases in our local operational data aggregation system—a health information exchange (HIE) environment, into which different data sources are integrated. The 4 use cases included health information exchange record deduplication (labeled as Indiana Network for Patient Care [INPC]), linkage of a public health registry Marion County Health Department records to HIE (labeled as MCHD), linkage of Social Security Death Master File records of the Social Security Administration to HIE (labeled as SSA), and deduplication of newborn screening records (labeled as NBS). We hypothesize that proper treatment of missing data and data-driven matching field selection will enhance linkage performance.

## Methods

### Blocking

Records need to be compared in record linkage to ascertain whether they belong to the same entity. Forming record pairs by Cartesian product from the 2 files (or to a file itself in the case of deduplication) results in an enormously large number of pairs. For example, the data set from the INPC (the INPC use case) has 47,334,986 records (Table 1) and will form 2.24 quadrillion record pairs by the Cartesian product. A common strategy is “blocking on” certain fields (blocking variables) to reduce the number of record pairs; that is, retaining only those record pairs with exact agreement in blocking variables. Blocking helps to enrich matches by restricting the search space. We applied 5 blocking schemes to each use case. In the INPC use case, the five blocking schemes are the Social Security number (SSN); first name and telephone number (FN-TEL); day, month, and year of birth and zip code (DB-MB-YB-ZIP); first name, last name, and year of birth (FN-LN-YB); and day, month, and year of birth and last name (DB-LN-MB-YB). These five blocking schemes contained 613 million record pairs, with the number of pairs in each block listed in Table 1. Within each block, record pairs are compared field by field for a collection of matching fields, yielding a vector of comparison results for each pair. For example, if only 3 matching fields are compared by exact comparison (for agreement or disagreement), the vectors will have 2^3^ possible patterns when there are no missing data. In general, if *K* matching fields are compared, there will be 2*^K^* total agreement patterns.

**Table 1:** Summary of four use cases, Indiana Network for Patient Care (INPC), newborn screening (NBS), Social Security Administration (SSA), and Marion County Health Department (MCHD), with information on the number of records in each use case, blocking schemes, and the numbers of record pairs in blocking schemes.

Block		Pairs
**INPC (47,334,986 records)**		
	SSN^a^	53,054,690
	FN-TEL^b^	41,729,402
	DB-MB-YB-ZIP^c^	133,553,036
	FN-LN-YB^d^	193,865,283
	DB-LN-MB-YB^e^	191,181,498
**NBS (765,813 records)**		
	MRN^f^	4,147,098
	TEL^g^	2,644,454
	MB-DB-ZIP	8,083,396
	LN-FN^h^	3,005,368
	NK_LN-NK_FN^i^	1,217,736
**SSA (89,556,520 records)**		
	SSN	805,331
	FN-LN-ZIP	18,103
	FN-LN-MI-YB	1,395,395
	FN-LN-MI-DB-MB	547,376
	FN-LN-DB-MB-YB	722,167
**MCHD (471,298 records)**		
	SSN	869,454
	TEL	28,238
	DB-MB-YB-zip	5,083,429
	FN-LN-YB	3,378,017
	DB-LN-MB-YB	3,701,460

^a^SSN: Social Security number.

^b^FN-TEL: first name and telephone number.

^c^DB-MB-YB-ZIP: day, month, and year of birth and zip code.

^d^FN-LN-YB: first name, last name, and year of birth.

^e^DB-LN-MB-YB: day, month, and year of birth and last name.

^f^MRN: medical record number.

^g^TEL: telephone no.

^h^LN-FN: last name, first name.

^i^NK_LN, NK_FN: next of kin last name and first name.

### The FS Model

Formally, for the *i*th pair of records, let *δ_i_* denote the unobserved true match status (a latent binary class variable) with a value of 1 indicating a match and 0 indicating a nonmatch (ie, the class label for match and nonmatch classes), *Y_i_*=(*Y_i_*_1_, … ,*Y_iK_*) be the vector of agreements in K fields, and y*_i_*=(y*_i_*_1_, … ,y*_iK_*) be the observed agreements. In addition, let *n* be the total number of pairs and *ρ=P(δ=1)*, the *match prevalence* in the total n pairs of records. Assuming independent observations *(y_i_, δ_i_), i=1,...,n*, we express its complete data likelihood and the marginal distribution of *y_i_, i=1,...,n* as follows:


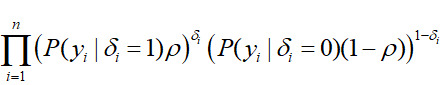
 and 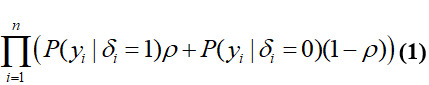. For a given *i*, the posterior probability of *δ_i_=1* is 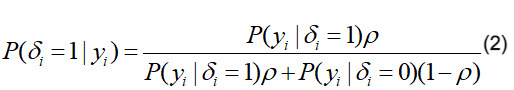. If the true match status *δ_i_*’s are known, then the MLE of *ρ* for the complete data likelihood is 
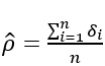
. When *δ_i_*’s are unknown, this problem is known as the latent class modeling because the model parameters are estimated without the class label being observed.

A popular algorithm, named after Fellegi and Sunter [1] in the probabilistic record linkage literature, further assumes 
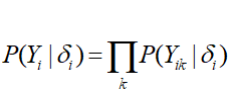
; that is, the assumption of conditional independence of *Y_i_*_1_, ... ,*Y_iK_* within each latent class. The FS model greatly simplifies the estimation process, producing estimates for field-specific probability of agreement given that a pair is a match, *m_k_=P(Y_ik_=1|δ_i_=1)*, and the field-specific probability of agreement given that a pair is a nonmatch, *u_k_=P(Y_ik_=1|δ_i_=0)*. Model estimates can be obtained by using the Expectation-Maximization (EM) algorithm [16] on the complete data likelihood or by using standard optimization routines on the marginal likelihood. The FS approach allows the model parameters to be estimated based on the observed agreements of pairs *without* the use of a training set, qualifying it as an unsupervised learning algorithm.

### Classification of Record Pairs

Match scores are defined as the logarithm of likelihood ratios, 
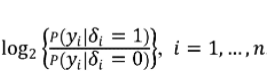
. Under the conditional independence assumption, the match score for the *i*th pair is the sum of the logarithms of the field-specific likelihood ratios, 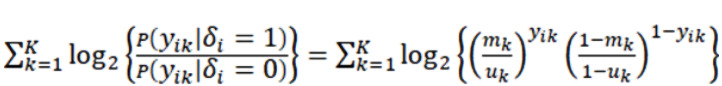. Match scores are computed using the estimated *m_k_* and *u_k_* from the FS model and are in turn used to rank all record pairs, with a high score indicating a higher likelihood of a record pair to be a match. In our study, we used the estimated match prevalence *ρ* to set the threshold as the upper *ρ*-th quantile of the scores. A record pair is then declared as a match if its score is greater than the threshold; otherwise, it is declared as a nonmatch.

### Treatment of Missing Data

Formally describing the missing data mechanism is important for devising an approach to account for missing data. Missing data are generally classified into 3 types [17]. First, the most restrictive type of missing data is missing completely at random (MCAR), which assumes that the missingness in a variable is independent of all observed or unobserved variables. In this situation, the parameter estimates are unbiased when record pairs with any missing data are excluded. However, omitting missing data may lower the precision of estimated parameters due to the smaller sample size. In addition, MCAR is a strong assumption that cannot be verified with the data at hand. Second, MAR is a less-restrictive yet more realistic missing data model that assumes that the missingness in a variable is independent of unobserved data, although it can depend on other observed variables. Finally, missing not at random (MNAR) asserts that the missingness of a variable is related to the unobserved variable itself. To handle MNAR, knowledge of the missing mechanism is required to model the missing process in the estimation of the parameters and matching scores.

In record linkage applications, missing values in matching fields are typically handled by excluding records with missing values on one of the matching fields when estimating match weights [5] or considering the field’s agreement pattern as a disagreement [6]. Excluding records with missing values is justifiable only when the data are MCAR. Thus, excluding records when the MCAR assumption does not hold leads to inaccurate results due to bias and low precision; the bias arises from the wrong model assumption and the low precision from the reduced sample size. Alternatively, treating missing data as disagreement (MAD) is implicitly invoking the assumption of MNAR, which may yield inaccurate results when the MAD assumption that all missing data represent disagreement is incorrect. This strong assumption is likely false for data to be linked. For example, if the middle name is absent because it does not exist, a missing value from both records of the record pair can provide information that the 2 records belong to the same person. On the other hand, the assumption of MAR is the least restrictive among the 3 types of missing mechanisms, and we hypothesize that it will yield superior match performance. Assuming MAR, the missing data are handled using the full information likelihood approach that uses all available data (ignoring the matching fields with missing values) in the FS model under the assumption of conditional independence of the matching fields.

The predictive results are obtained the same way for the FS model with MAR and MAD. The difference lies in the manner in which missing data are treated. When MAD is used, fields with missing data are set to “disagreement” (coded as 0), and the FS algorithm as is can proceed on the data with missing values replaced by zeros. When MAR is used, the FS algorithm is used on nonmissing data. In either cases, parameters *m_k_*,*u_k_* and the match prevalence are estimated, and match scores are calculated for all pairs. The threshold for a pair to be a match is set to be the upper -th quantile of the scores. A record pair is then declared as a match if its score is greater than the threshold; otherwise, it is declared a nonmatch.

### Selection of Matching Fields

Fields missing 100% within a blocking scheme contain no information and will not be considered further. We examined 2 approaches selecting matching fields: the standard practice of subject matter expert-guided field selection and a data-driven approach. In the data-driven approach, all fields were considered to be putative matching fields. A necessary condition for a field to be useful in matching is that it should exhibit variability. For example, if the value of a field is fixed (no variation), it cannot separate matches from nonmatches. Thus, a blocking variable can no longer be used as a matching field in a block formed using the blocking variable. When running an FS model, we started with the largest possible set of fields; more fields may be dropped from the model, starting with fields with the least variations, until the FS algorithm converges.

### Data Sets of 4 Use Cases and Gold Standards

We evaluated the matching performance of the missing data treatment (MAD and MAR) and matching field selection (expert-specified fields vs data-driven fields) by conducting a 2-by-2 factorial design using 4 real-world use cases in our local HIE environment. The 4 use cases contain data that were generated as part of clinical or public health processes.

The 4 use cases included deduplicating clinical records in a state-level HIE, linking a public health registration file to clinical data in the HIE, linking death records to clinical data in the HIE, and deduplication of the Health Level Seven International (HL7) messages for newborns less than 1 month of age from the HIE. For each use case, blocking was performed to confine the total number of record pairs to be compared with a subspace of record pairs enriched with true matches [18]. Five blocking schemes were selected for each use case based on expert input from our laboratory. The total number of records for each use case and the number of record pairs per blocking scheme are listed in Table 1. To assess the matching performance, we selected record pairs for human review by performing proportional sampling from the union of record pairs with strata defined by the five blocking schemes. To compare the sensitivity of the algorithms, a total of 5884 true matches were necessary to test a 2% absolute difference in discordant rates of the 2 algorithms among the true matches with an 80% power at a 2-sided significance level of .05. The same number of true nonmatches was required to test the 2% difference in specificity. We sampled record pairs until we reached at least 5884 pairs in the class of true matches and the class of true nonmatches. Each record pair was reviewed by 2 reviewers; in the case of a disagreement in the classification of the pair, a third reviewer adjudicated the pair. Table 2 summarizes the manually reviewed sets for the 4 use cases.

**Table 2:** Manual review results for the 4 use cases.

Use case	Number of pairs^a^	Number of pairs deemed as matches	Number of pairs deemed as nonmatches	Match prevalence^b^
INPC^c^	15,000	7840	7160	0.523
SSA^d^	16,500	5950	10,550	0.361
NBS^e^	15,000	7967	7033	0.531
MCHD^f^	15,500	5927	9573	0.382

^a^Number of pairs is the total number of pairs sampled for manual review, which determines the pairs as either matches or nonmatches.

^b^Match prevalence is the ratio of the number of pairs deemed as matches and the total number of pairs for manual review for each use case.

^c^INPC: Indiana Network for Patient Care.

^d^SSA: Social Security Administration.

^e^NBS: newborn screening.

^f^MCHD: Marion County Health Department.

#### Deduplicating HIE (INPC)

This data set reflected demographic records from geographically proximal hospital systems that participate in HIE. Blocking is as described earlier. The data contained a subset of 15,000 sampled gold standard pairs with 7840 (52.3%) true positives and 7160 (47.7%) true negatives. Patients from hospitals in close proximity cross over to nearby institutions, creating the need to identify common records. New value-based purchasing models such as Accountable Care Organizations dramatically increased the need to identify and capture information on patients seeking care from other institutions.

#### HIE and Vital Records for Ascertaining Death Status (SSA)

These data reflect a combination of the Social Security Death Master File and HIE data. We applied five blocking schemes (Table 1): SSN; first name, last name, and zip code (FN-LN-ZIP); first name, last name, middle initial, and year of birth (FN-LN-MI-YB); first name, last name, middle initial, and day and month of birth (FN-LN-MI-DB-MB); and first name, last name, and day, month, and year of birth (FN-LN-DB-MB-YB). This data set contained a subset of 16,500 sampled gold standard pairs with 5950 (36.1%) true positives and 10,550 (63.9%) true negatives. Accurately and comprehensively updating health records with patients’ accurate death status is critical for robust clinical quality measurement, public health reporting requirements, and high-quality clinical research.

#### Deduplicating Newborn Registration Data (NBS)

This data set included demographic data for newborns derived from multiple hospitals, clinics, and within the HIE. These data were limited to patients aged <2 months. We applied five blocking schemes (Table 1): medical record number (MRN), telephone number (TEL), month, day of birth, zip code (MB-DB-ZIP), last name and first name (LN-FN), and next of kin’s last name and first name (NK_LN-NK_FN). This data set contained a subset of 15,000 sampled gold standard pairs, with 7967 (53.1%) true positives and 7033 (46.9%) true negatives. Matching in this cohort is important because not all infants receive appropriate screening for harmful or potentially fatal disorders that are otherwise unapparent at birth [4]. Public health screening tests must be linked to patient records to avoid harmful delays in diagnosis.

#### Public Health Registry Linked to Clinical Registrations (MCHD)

This data set comes from the MCHD, Indiana’s largest public health department. The registry contains a master list of demographic information for clients who receive public health services such as immunization; Women, Infants, and Children’s nutrition support; and laboratory testing [19,20]. The registry also tracks population health trends and supports other public health activities. Duplicate patient records are often unintentionally added. We applied five blocking schemes (Table 1): SSN; telephone number (TEL); day, month, and year of birth and zip code (DB-MB-YB-ZIP); first name, last name, and year of birth (FN-LN-YB); and day, month, and year of birth and last name (DB-LN-MB-YB). This data set contained a subset of 15,500 sampled gold standard pairs with 5927 (38.2%) true positives and 9573 (61.8%) true negatives. We linked the complete patient registry to patient records in the aforementioned HIE.

The 4 data sets contained subsets of the following fields: MRN, SSN, last name (LN), first name (FN), middle initial (MI), nickname (NICK_SET), ethnicity (ETH_IMP), sex, month of birth (MB), day of birth (DB), YB, street address (ADR), city, state (ST), zip code (ZIP), telephone number (TEL), email, last name of next of kin (NK_LN), first name of next of kin (NK_FN), last name of treating physician (DR_FN), and first name of treating physician (DR_LN). The last 4 fields were used only in the NBS use case.

### Analyses of Use Cases

For each use case, blocking was performed first, and five blocks of record pairs were generated. The blocking schemes are listed in Table 1. The FS model is applied 4 times in each block based on the 2-by-2 factorial design, where missing data are either treated using MAD or MAR, and matching fields are either expert-specified or selected by the data-driven method. The parameters of the FS model can be estimated using the Newton Raphson approach or the EM algorithm, both of which maximize the likelihood function of the model. The exact agreement on the following fields (when available for a use case) was considered in the matching in INPC, MCHD, and SSA use cases: street address, city (in address), DB, MB, YB, EMAIL, ethnicity, FN, LN, MI, MRN, nickname, sex, state, ZIP, and TEL. MCHD and SSA do not have MRN; all 4 use cases include the nickname and ethnicity as derived fields. The fields used in the NBS use case were slightly different. The exact agreement on the following fields was considered in the matching in the NBS use case: street address, city, DB, MB, YB, physician’s FN, physician’s LN, email address, ethnicity, FN, LN, MI, nick name, MRN, NK_FN, NK_LN, sex, SSN, state, TEL, and ZIP. The fields of exact agreement available for matching and their percentages of missing values are summarized in Multimedia Appendices 1-4 for the 4 use cases.

Within each run of the FS model, the estimate of block-specific prevalence under each missing treatment was used to classify record pairs as matches and nonmatches (see Classification of Record Pairs); the union of matches from all 5 blocks is the set of matches obtained.

### Evaluation of Record Linking Performance

To evaluate the accuracy of these matching models, we calculated the following metrics: sensitivity, specificity, positive predictive value (PPV), negative predictive value (NPV), and *F*_1_-score, as well as their respective 95% CIs based on 999 bootstrap samples. The abovementioned metrics were estimated from gold standard sets for which manual reviews established the true match status. Our primary evaluation metric was the *F*_1_-score.

### Ethics Approval

This study was reviewed and approved by the Indiana University Institutional Review Board (IRB#: 1703755361).

## Results

The 4 use cases contained missing data to various extents. Notably, 45.8% of the 47,334,986 records in the INPC use case had no SSN, making it necessary to add other blocking schemes that do not rely on the SSN. For the NBS use case, the SSN is typically missing because infants do not receive an SSN for at least 2 to 6 weeks after birth and often later if parents do not initially request the identifier. When linking the other 2 use cases SSA and MCHD to INPC, due to the INPC data set missing SSN in 45.8% of its records, blocking on SSN alone yielded only 4547 out of 5950 (76%) and 1531 out of 5927 (26%) of true matches in SSA and MCHD, respectively, based on the manually reviewed subsets (Multimedia Appendices 5 and 6).

Additional blocking schemes are essential to increase match sensitivity. As the FS algorithm is performed using paired data per blocking scheme, its performance is directly affected by the extent of missing values in the agreement vectors obtained by comparing pairs of records within each block. We summarized the proportions of missing data in the 5 blocking schemes of each use case in Multimedia Appendices 1-4. The proportion of missing values per block ranges from 0% to 100%. Fields that were missing 100% within a blocking scheme contained no information and, therefore, were not considered further. The extent of missing values in a matching field does not necessarily negatively correlate with the discriminating power of the field.

Matching fields with even substantial missing values nonetheless proved to be useful in discriminating matches from nonmatches. For example, the agreement status of email address comparison is missing for 99% of record pairs in the DB-LN-MB-YB blocking scheme of the INPC use case; the m- and u-probabilities were estimated to be 0.01147 and 0.000204 under MAD and 0.3830 and 0.02553 under MAR, respectively. The large ratios of the m-probability over the u-probability in either case indicate the utility of email address in linkage. As another example, the agreement status of zip code comparison is also missing for 99% of record pairs in the FN-LN-MI-YB block of the SSA use case, and the m- and u-probabilities were estimated to be 0.02073 and 8.49×10^−7^ under MAD and 0.7538 and 0.000137 under MAR, respectively. In both examples, the estimates of m- and u-probabilities are much larger under MAR than under MAD, suggesting that a downward bias might be incurred by artificially setting missing values to disagreement in the MAD approach.

The fields used by the final FS models, either expert-specified or data-driven per block per use case, are summarized in Table 3. Except for the SSA use case, where the number of fields that could be used for matching is limited, the number of data-driven fields is greater than the number of expert-specified fields for the remaining 3 use cases.

**Table 3:** Summary of modeling information by data use case and by blocking scheme.

Data and block		Expert-specified fields^a^	Data-driven fields^a^
**INPC**			
	DB-LN-MB-YB^b^	MRN^c^ FN^d^ SEX^e^ TEL^f^ ADR^g^ ZIP^h^ SSN^i^	MRN FN SEX TEL ADR ZIP SSN *CITY EMAIL ETH MI NICK ST*^j^
	DB-MB-YB-ZIP	MRN LN FN SEX TEL ADR SSN	MRN LN FN SEX TEL ADR SSN *CITY EMAIL ETH MI NICK ST*
	FN-LN-YB	MRN SEX DB MB TEL ADR ZIP SSN	MRN SEX DB MB TEL ADR ZIP SSN *CITY EMAIL MI ST ETH NICK*
	FN-TEL	MRN LN SEX DB MB YB ADR ZIP SSN	MRN LN SEX DB MB YB ADR ZIP SSN *CITY EMAIL ETH MI NICK ST*
	SSN	MRN LN FN SEX DB MB YB TEL ADR ZIP	MRN LN FN SEX DB MB YB TEL ADR ZIP *CITY EMAIL ETH MI NICK ST*
**SSA^k^**			
	FN-LN-DB-MB-YB	SSN MI ZIP	SSN MI ZIP
	FN-LN-MI-DB-MB	ZIP YB SSN	ZIP YB SSN
	FN-LN-MI-YB	DB MB ZIP SSN	DB MB ZIP SSN
	FN-LN-ZIP	MI DB MB YB SSN	MI DB MB YB SSN
	SSN	LN FN MI DB MB YB ZIP	LN FN MI DB MB YB ZIP
**NBS^l^**			
	LN-FN	MRN SEX DB MB YB TEL ADR ZIP	MRN SEX^m^ DB MB YB^m^ TEL ADR ZIP *CITY DR_FN DR_LN MI NK_FN NK_LN*
	MB-DB-ZIP	MRN LN FN SEX YB TEL ADR	MRN LN FN SEX YB TEL ADR *CITY DR_FN DR_LN ETH MI NK_FN NK_LN NICK*
	MRN	LN FN SEX DB MB YB TEL ADR ZIP	LN FN SEX^m^ DB MB YB TEL^m^ ADR ZIP *CITY DR_FN DR_LN ETH MI NK_FN NK_LN ST*
	NK_LN-NK_FN	MRN LN FN SEX DB MB YB TEL ADR ZIP	MRN LN^m^ FN SEX DB MB YB TEL ADR ZIP *CITY DR_FN DR_LN ETH LN MI NICK ST*
	TEL	MRN LN FN SEX DB MB YB ADR ZIP	MRN LN FN SEX DB MB YB^m^ ADR ZIP *CITY DR_FN DR_LN ETH MI NK_FN NK_LN ST*
**MCHD^n^**			
	LN-FN	MRN SEX DB MB YB TEL ADR ZIP	MRN SEX^m^ DB MB YB^m^ TEL ADR ZIP *CITY DR_FN DR_LN MI NK_FN NK_LN*
	MB-DB-ZIP	MRN LN FN SEX YB TEL ADR	MRN LN FN SEX YB TEL ADR *CITY DR_FN DR_LN ETH MI NK_FN NK_LN NICK*
	MRN	LN FN SEX DB MB YB TEL ADR ZIP	LN FN SEX^m^ DB MB YB TEL^m^ ADR ZIP *CITY DR_FN DR_LN ETH MI NK_FN NK_LN ST*
	NK_LN-NK_FN	MRN LN FN SEX DB MB YB TEL ADR ZIP	MRN LN^m^ FN SEX DB MB YB TEL ADR ZIP *CITY DR_FN DR_LN ETH LN MI NICK ST*
	TEL	MRN LN FN SEX DB MB YB ADR ZIP	MRN LN FN SEX DB MB YB^m^ ADR ZIP *CITY DR_FN DR_LN ETH MI NK_FN NK_LN ST*

^a^Columns “Expert-specified fields” and “Data-driven fields” display the fields used in the Fellegi-Sunter (FS) model.

^b^DB-LN-MB-YB: day, month, and year of birth and last name.

^c^MRN: medical record number.

^d^FN: first name.

^e^SEX: sex.

^f^TEL: telephone number.

^g^ADR: address.

^h^ZIP: zip code.

^i^SSN: Social Security number.

^j^Fields (italicized) selected only by data-driven methods.

^k^SSA: Social Security Administration.

^l^NBS: newborn screening.

^m^Fields not selected by the data-driven method but specified by experts.

^n^MCHD: Marion County Health Department.

The matching metrics of the 4 use cases evaluated on their respective ground truth sets of randomly selected and manually reviewed record pairs are displayed in Table 4. Multimedia Appendices 5-7. From Table 4, we observe the following:

1. MAR improves the *F1*-score in general, whether matching fields are expert-specified or data-driven; the improvement in the *F1*-score comes from improved sensitivity with comparable or better PPV. The largest improvement in the *F1*-score occurred in the NBS use case, 0.874 with MAR using data-driven fields compared with 0.837 with MAD using expert-specified fields.
2. MAD using expert-specified fields had higher *F1*-scores than *F1*-scores using data-driven fields (except for NBS). As the number of data-driven fields is usually greater than the number of expert-specified fields in a block, we hypothesized that the artificial correlations among the large number of data-driven fields induced by MAD adversely affect the match performance.
3. MAR coupled with data-driven fields yielded *F1*-scores comparable to or larger than those of MAR with expert-specified fields and larger than the F1-scores of MAD with both methods of field selection.

In the SSA use case, the *F*_1_-scores of both methods were similar, 0.873 for MAD and 0.875 for MAR, with either expert-specified matching fields or data-driven matching fields, because both field-selection approaches selected the same set of matching fields. We examined the classification results within the classes of true matches and true nonmatches in the ground truth set, on whether the classified matches and nonmatches were similar or whether the 2 methods made different mistakes. From the diagonals in Table 5, we can see that the 2 methods produce roughly congruent classification results in the classes of true matches and true nonmatches. FS under MAR is slightly more sensitive than FS under MAD: 26 true matches that are misclassified as nonmatches by MAD are recovered as matches by MAR; only 3 nonmatches are misclassified as matches by MAR, but 1 nonmatch that is misclassified as a match by MAD is correctly classified as a nonmatch by MAR. In summary, the classification results are similar in the SSA use case; FS under MAR is slightly more sensitive than FS under MAD, while maintaining PPV, NPV, and specificity (Table 4).

The algorithms performed differently, partly because of the different data quality of the use cases. The *F*_1_-score was 0.979 for INPC but only 0.874 for NBS (Table 4). First, INPC and NBS use cases have very different patterns of missing data across matching fields; for example, SSN is missing from 52.6% to 69.7% of record pairs (except for the SSN block, which by definition has no missing SSN) across the 5 blocks in INPC, whereas SSN is missing in more than 98% of record pairs in NBS (Multimedia Appendices 1 and 3). Second, the discriminating powers of the same fields were different in the 2 use cases. A field has high discriminating power if its agreement rate is high among matches and low among nonmatches; otherwise, the data quality is indicated as low. For example, the probabilities of agreement in the fields of the LN and FN in the same DB-MB-YB and ZIP blocking scheme for INPC and NBS show differential data quality: 94.45% of matches of INPC agree on the LN, while only 89% of matches of NBS do; on the other hand, only 0.45% of nonmatches of INPC agree on the LN but 2.70% of nonmatches of NBS do; 94.36% of matches of INPC agree on the FN, while only 66.99% of matches of NBS do; only 0.40% of nonmatches of INPC agree on the FN but 3.45% of nonmatches of NBS do (Multimedia Appendix 8). As the matching score is a summation of the ratios of the agreement probabilities of the matches versus the nonmatches on the log-scale across all matching fields, data quality directly affects matching performance.

**Table 4:** Matching results of the four use cases evaluated on their respective ground truth sets of random-selected and manually reviewed record pairs.

Data				Value, N		Sensitivity (95% CI)		Specificity (95% CI)		Positive predictive value (95% CI)		Negative predictive value (95% CI)		*F*_1_-score (95% CI)
**Expert-specified fields**														
	**INPC^a^**													
		MAD^b^	15,000		0.962 (0.958-0.967)		0.990 (0.987-0.992)		0.990 (0.988-0.992)		0.960 (0.955-0.964)		0.976 (0.974-0.978)	
		MAR^c^	15,000		0.970 (0.966-0.974)		0.988 (0.986-0.991)		0.989 (0.987-0.991)		0.968 (0.964-0.972)		0.980 (0.977-0.982)	
	**SSA^d^**													
		MAD	16,500		0.781 (0.770-0.792)		0.995 (0.994-0.996)		0.989 (0.986-0.992)		0.890 (0.884-0.895)		0.873 (0.866-0.879)	
		MAR	16,500		0.785 (0.775-0.796)		0.995 (0.993-0.996)		0.989 (0.985-0.991)		0.892 (0.886-0.897)		0.875 (0.869-0.882)	
	**NBS^e^**													
		MAD	15,000		0.795 (0.786-0.804)		0.881 (0.874-0.889)		0.883 (0.876-0.891)		0.791 (0.782-0.801)		0.837 (0.830-0.843)	
		MAR	15,000		0.860 (0.852-0.868)		0.873 (0.865-0.881)		0.885 (0.877-0.892)		0.846 (0.838-0.855)		0.872 (0.866-0.878)	
	**MCHD^f^**													
		MAD	15,500		0.944 (0.937-0.949)		0.989 (0.987-0.991)		0.982 (0.979-0.986)		0.966 (0.962-0.969)		0.963 (0.959-0.966)	
		MAR	15,500		0.946 (0.940-0.952)		0.988 (0.986-0.990)		0.980 (0.976-0.983)		0.967 (0.964-0.971)		0.963 (0.959-0.966)	
**Data-driven fields**														
	**INPC**													
		MAD	15,000		0.579 (0.568-0.590)		0.988 (0.986-0.991)		0.982 (0.978-0.985)		0.682 (0.672-0.690)		0.729 (0.719-0.737)	
		MAR	15,000		0.970 (0.966-0.974)		0.987 (0.984-0.989)		0.988 (0.985-0.990)		0.968 (0.964-0.972)		0.979 (0.976-0.981)	
	**SSA**													
		MAD	16,500		0.781 (0.770-0.792)		0.995 (0.994-0.996)		0.989 (0.986-0.992)		0.890 (0.884-0.895)		0.873 (0.866-0.879)	
		MAR	16,500		0.785 (0.775-0.796)		0.995 (0.993-0.996)		0.989 (0.985-0.991)		0.892 (0.886-0.897)		0.875 (0.869-0.882)	
	**NBS**													
		MAD	15,000		0.813 (0.805-0.822)		0.875 (0.867-0.883)		0.880 (0.873-0.888)		0.805 (0.796-0.814)		0.845 (0.839-0.852)	
		MAR	15,000		0.865 (0.858-0.873)		0.870 (0.863-0.878)		0.883 (0.876-0.890)		0.851 (0.842-0.859)		0.874 (0.868-0.880)	
	**MCHD**													
		MAD	15,500		0.635 (0.622-0.648)		0.970 (0.967-0.974)		0.929 (0.921-0.937)		0.811 (0.804-0.818)		0.754 (0.745-0.764)	
		MAR	15,500		0.954 (0.948-0.959)		0.988 (0.985-0.990)		0.979 (0.976-0.983)		0.972 (0.968-0.975)		0.967 (0.963-0.970)	

^a^INPC: Indiana Network for Patient Care.

^b^MAD: missing as disagreement.

^c^MAR: missing at random.

^d^SSA: Social Security Administration.

^e^NBS: newborn screening.

^f^MCHD: Marion County Health Department.

**Table 5:** Cross-tabulation of ground truth and classification results by the Fellegi-Sunter model under missing as disagreement (MAD) and missing at random (MAR) for the Social Security Administration use case.

MAD		MAR		Values, N
		Nonmatch	Match	
**Man_Rev_Status: matches**				
	Nonmatch	1277	26	1303
	Match	0	4647	4647
	Value, N	1277	4673	5950
**Man_Rev_Status: nonmatches**				
	Nonmatch	10,495	3	10,498
	Match	1	51	52
	Value, N	10,496	54	10,550

## Discussion

The US health care system will likely not have a unique and universal patient ID in the near future, so innovations such as incorporating missing data under MAR and data-driven field selection in the linkage algorithms are necessary to optimize existing methods to ensure accurate patient identity and support patient safety. Our findings are important because they demonstrate improvements in linkage performance among 4 different but representative use cases. Our HIE-based patient-matching laboratory has experience matching clinical data from heterogeneous sources, including hospitals (inpatient and emergency departments) [21,22], ambulatory care settings [23], public health syndrome as captured by surveillance systems [24], electronic laboratory reporting in case detection systems [25], and NBS data [26]. Thus, we can specifically measure variations in patient-matching performance across different use cases for the same patient-matching approaches. Through this work, we have shown that the performance of the 2 enhancements in patient-matching algorithms may have additive values across different clinical use cases.

Although the assumption of missing at random is not verifiable, the success of the FS algorithm coupled with MAR in our four different use cases indicates that missing at random is a reasonable assumption. As MCAR is a special case of MAR, our algorithm works when data are MCAR. These results will inform future research and development in patient-matching spaces.

Furthermore, the superior performance observed with MAR using data-driven fields over other combinations in the 2×2 design and four use cases suggests its potential value for incorporation into privacy-preserving record linkage (PPRL) methods. In PPRL, to preserve privacy, fields can be tokenized (eg, using bigrams) into smaller parts and compared [27]. As the tokens do not reveal the actual nature and content of the field, an expert cannot specify matching fields as they can with fields such as names, DB, SSN, etc. PPRL is a scenario in which data-driven field selection coupled with MAR in the FS model appears useful.

Finally, many data-driven fields may lead to model overfitting, which is a prominent cause of the poor performance of machine learning algorithms. In many applications in medical research using latent class models, many covariates are available, and the number of covariates overwhelms the number of observations. This is the main motivation for most of the variable selection literature to identify a subset of variables to (1) estimate the association between the covariates and the response variable and (2) obtain a parsimonious model that describes the covariates and the response variable [28]. However, in record linkage, the primary concern is not estimation but rather the prediction of the *unknown* response variable—the class label of match or nonmatch, model parsimony is irrelevant. Sample sizes in record linkage are generally large: in our four use cases, even the sample size of the smaller class of our smallest use case, MCHD, is approximately 180,035 (Tables 1 and 2). Hence, the number of matching fields used relative to the overall samples and the within-class sample sizes were small, without concern for overfitting. The FS model for record linkage is an unsupervised algorithm in that the response variable indicates whether a pair of records belonging to the same entity is unknown and is to be inferred. As such, the veracity of any unsupervised classification algorithm applied to a set of record pairs is tested on a representative ground truth set in which the class labels are obtained through manual reviews. As described in the Methods section, we created a gold standard set of randomly selected record pairs for each of the 4 use cases. Most importantly, the match status of those record pairs in the gold standard sets was not used in the FS model fitting process (including field selection). As the data-driven fields used in the FS model under MAR uniformly perform the best for all four use cases, overfitting is not a concern because overfitting tends to hurt performance.

While we strive to generate results that are applicable to the broadest possible audience using a health informatics research laboratory that captures a diverse set of data elements with varying data characteristics, we cannot assure generalizability with complete certainty. If our data are not representative of other health systems, then our linkage results may not be applicable. If the missing data mechanism is not MAR or MCAR (eg, if the missingness of a data element is related to its value), our algorithm will likely not work. Before applying our methods to a data environment with missing data, we recommend creating a ground truth set of randomly selected record pairs whose match status is manually reviewed to determine whether our methods are applicable to a specific data environment.

Finally, our results suggest that accommodating missingness in patient-matching algorithms can improve accuracy. While the FS model is widely used, different FS implementations and completely different models (eg, decision trees or boosting algorithms) may exhibit a greater or lesser effect. We will explore the potential of these machine learning tools in our future work.

In summary, the combination of data-driven matching field selection and MAR methods produced the best overall performance for four real-world matching use cases. The MAR method maintained or improved *F*_1_-scores regardless of whether matching fields were expert-specified or data-driven, suggesting that MAR is a reasonable assumption for patient-record linkage in real-world settings. As the implementation of MAR requires minimal effort and improves or maintains linkage accuracy, we advocate using this approach over MAD in record linkage, provided that adequate evaluation using manually reviewed data is performed to ensure method generalizability to a specific data environment. These methods can be useful for PPRL, where expert field selection may not be possible.

## Appendix

Multimedia Appendix 1 Table S1 Proportion of missing values by matching field in the Indiana Network for Patient Care (INPC) use case. For each blocking scheme (column) the unshaded fields are used for matching in the final Fellegi-Sunter (FS) model for that block in the data-driven approach.

Multimedia Appendix 2 Table S2 Proportion of missing values by field in the Social Security Administration (SSA) use case. For each blocking scheme (column) the unshaded fields are used for matching in the final Fellegi-Sunter (FS) model for that block in the data-driven approach.

Multimedia Appendix 3 Table S3 Proportion of missing values by field in the newborn screening (NBS) use case. For each blocking scheme (column) the unshaded fields are used for matching in the final Fellegi-Sunter (FS) model for that block in the data-driven approach.

Multimedia Appendix 4 Table S4 Proportion of missing values by field in the Marion County Health Department (MCHD) use case. For each blocking scheme (column) the unshaded fields are used for matching in the final Fellegi-Sunter (FS) model for that block in the data-driven approach.

Multimedia Appendix 5 Table S5 Matching results of the SSA use case evaluated on a set of 16,500 randomly selected and manually reviewed record pairs. The first two rows are the overall results combined from all blocks on the manually reviewed sample, with the first row for MAD (missing as disagreement) and the second row for MAR (missing at random). Every subsequent two rows pertain to a specific block, with the first containing the results of MAD and the 2nd row the results of MAR. Columns N, SEN, SPE, PPV, NPV and F1 are the total number of manually reviewed record pairs, sensitivity, specificity, positive predictive value, negative predictive value and F-score.

Multimedia Appendix 6 Table S6 Matching results of the newborn screening (NBS) use case evaluated on a set of 15,000 randomly selected and manually reviewed record pairs. The first two rows are the overall results combined from all blocks on the manually reviewed sample, with the first row for MAD (missing as disagreement) and the second row for MAR (missing at random). Every subsequent two rows pertain to a specific block, with the first containing the results of MAD and the 2nd row the results of MAR. Columns N, SEN, SPE, PPV, NPV, and F1 are the total number of manually reviewed record pairs, sensitivity, specificity, positive predictive value, negative predictive value and F-score.

Multimedia Appendix 7 Table S7 Matching results of the Marion County Health Department (MCHD) use case evaluated on a set of 15,500 randomly selected and manually reviewed record pairs. The first two rows are the overall results combined from all blocks on the manually reviewed sample, with the first row for MAD (missing as disagreement) and the second row for MAR (missing at random). Every subsequent two rows pertain to a specific block, with the first containing the results of MAD and the 2nd row the results of MAR. Columns N, SEN, SPE, PPV, NPV, and F1 are the total number of manually reviewed record pairs, sensitivity, specificity, positive predictive value, negative predictive value and F-score.

Multimedia Appendix 8 Table S8 Data quality of fields of last name and first name in the DOB-ZIP block of the Indiana Network for Patient Care (INPC) and newborn screening (NBS) use cases.

## Abbreviations

CITY: city
DB: day of birth
EM: Expectation-Maximization
EMR: electronic medical record
ETH: ethnicity
FN: first name
FS: Fellegi-Sunter
HIE: health information exchange
HL7: Health Level Seven International
INPC: Indiana Network for Patient Care
LN: last name
LN-FN: last name and first name
MAR: missing at random
MB: month of birth
MCAR: missing completely at random
MCHD: Marion County Health Department
MI: middle initial
MNAR: missing not at random
MRN: medical record number
NBS: newborn screening
NPV: negative predictive value
PPRL: privacy-preserving record linkage
PPV: positive predictive value
SSA: Social Security Administration
SSN: Social Security number
YB: year of birth

## References

[1] Fellegi IP, Sunter AB (1969). A theory for record linkage. J Am Stat Assoc.

[2] Grannis SJ, Overhage JM, Hui S, McDonald CJ (2003). Analysis of a probabilistic record linkage technique without human review. AMIA Annu Symp Proc.

[3] Culbertson A, Goel S, Madden M, Safaeinili N, Jackson K, Carton T, Waitman R, Liu M, Krishnamurthy A, Hall L, Cappella N, Visweswaran S, Becich M, Applegate R, Bernstam E, Rothman R, Matheny M, Lipori G, Bian J, Hogan W, Bell D, Martin A, Grannis S, Klann J, Sutphen R, O’Hara A, Kho A (2017). The building blocks of inter-operability. Appl Clin Inform.

[4] Tromp M, Ravelli AC, Méray N, Reitsma JB, Bonsel GJ (2008). An efficient validation method of probabilistic record linkage including readmissions and twins. Methods Inf Med.

[5] Tromp M, Reitsma JB, Ravelli AC, Méray N, Bonsel GJ (2006). Record linkage: making the most out of errors in linking variables. AMIA Annu Symp Proc.

[6] Daggy JK, Xu H, Hui SL, Gamache RE, Grannis SJ (2013). A practical approach for incorporating dependence among fields in probabilistic record linkage. BMC Med Inform Decis Mak.

[7] Brown AP, Randall SM, Ferrante AM, Semmens JB, Boyd JH (2017). Estimating parameters for probabilistic linkage of privacy-preserved datasets. BMC Med Res Methodol.

[8] Allison P (2001). Missing Data.

[9] Donders AR, van der Heijden GJ, Stijnen T, Moons KG (2006). Review: a gentle introduction to imputation of missing values. J Clin Epidemiol.

[10] Jones MP (1996). Indicator and stratification methods for missing explanatory variables in multiple linear regression. J Am Stat Assoc.

[11] Knol MJ, Janssen KJ, Donders AR, Egberts AC, Heerdink ER, Grobbee DE, Moons KG, Geerlings MI (2010). Unpredictable bias when using the missing indicator method or complete case analysis for missing confounder values: an empirical example. J Clin Epidemiol.

[12] Vach W, Blettner M (2005). Missing data in epidemiologic studies. Encyclopedia of Biostatistics.

[13] Vink G, Frank LE, Pannekoek J, van Buuren S (2014). Predictive mean matching imputation of semicontinuous variables. Statistica Neerlandica.

[14] ENAMORADO T, FIFIELD B, IMAI K (2019). Using a probabilistic model to assist merging of large-scale administrative records. Am Polit Sci Rev.

[15] Xu H, Li X, Shen C, Hui SL, Grannis S (2019). Incorporating conditional dependence in latent class models for probabilistic record linkage: does it matter?. Ann Appl Stat.

[16] Dempster AP, Laird NM, Rubin DB (2018). Maximum likelihood from incomplete data via the algorithm. J Royal Statis Soc Series B (Methodological).

[17] Little R, Rubin D (2014). Statistical Analysis with Missing Data.

[18] Steorts R, Ventura S, Sadinle M, Fienberg S (2014). A comparison of blocking methods for record linkage. Privacy in Statistical Databases.

[19] Jain A, Flynn P, Ross A (2008). Handbook of Biometrics.

[20] Prabhakar S, Pankanti S, Jain A (2003). Biometric recognition: security and privacy concerns. IEEE Secur Privacy.

[21] Finnell J, Overhage J, Grannis S (2011). All health care is not local: an evaluation of the distribution of Emergency Department care delivered in Indiana. AMIA Annu Symp Proc.

[22] Wu J, Grannis SJ, Xu H, Finnell JT (2016). A practical method for predicting frequent use of emergency department care using routinely available electronic registration data. BMC Emerg Med.

[23] Overhage JM, Grannis S, McDonald CJ (2008). A comparison of the completeness and timeliness of automated electronic laboratory reporting and spontaneous reporting of notifiable conditions. Am J Public Health.

[24] Grannis S, Wade M, Gibson J, Overhage JM (2006). The Indiana public health emergency surveillance system: ongoing progress, early findings, and future directions. AMIA Annu Symp Proc.

[25] Gichoya J, Gamache RE, Vreeman DJ, Dixon BE, Finnell JT, Grannis S (2012). An evaluation of the rates of repeat notifiable disease reporting and patient crossover using a health information exchange-based automated electronic laboratory reporting system. AMIA Annu Symp Proc.

[26] Zhu VJ, Overhage MJ, Egg J, Downs SM, Grannis SJ (2009). An empiric modification to the probabilistic record linkage algorithm using frequency-based weight scaling. J Am Medical Inform Assoc.

[27] Schnell R, Bachteler T, Reiher J (2009). Privacy-preserving record linkage using Bloom filters. BMC Med Inform Decis Mak.

[28] Khalili A, Chen J (2012). Variable selection in finite mixture of regression models. J Am Statistical Assoc.

