# Supplementary material for: The Data-Adaptive Fellegi-Sunter Model for Probabilistic Record Linkage: Algorithm Development and Validation for Incorporating Missing Data and Field Selection
Source: J Med Internet Res. 2022 Sep 29;24(9):e33775. doi: 10.2196/33775 (PMC9562057; doi:10.2196/33775)
Supplement: Multimedia Appendix 1 [file jmir_v24i9e33775_app1.docx]

**Multimedia Appendix 1**

Table S1 Proportion of missing values by matching field in the INPC use case. For each blocking scheme (column) the ***unshaded*** fields are used for matching in the final FS model for that block in the data-driven approach.

| **Matching field** | **Blocking scheme** | | | | |
| --- | --- | --- | --- | --- | --- |
|  | **db-ln-mb-yb** | **db-mb-yb-zip** | **fn-ln-yb** | **fn-tel** | **ssn** |
| **MRN_agree** | 0.010 | 0.007 | 0.008 | 0.002 | 0.001 |
| **SSN_agree** | 0.697 | 0.526 | 0.688 | 0.631 | 0.000 |
| **LN_agree** | 0.000 | 0.000 | 0.000 | 0.000 | 0.000 |
| **FN_agree** | 0.000 | 0.001 | 0.000 | 0.000 | 0.001 |
| **Nick_agree** | 0.552 | 0.688 | 0.456 | 0.480 | 0.496 |
| **MI_agree** | 0.676 | 0.696 | 0.668 | 0.428 | 0.592 |
| **ETH_agree** | 0.000 | 0.000 | 0.000 | 0.000 | 0.000 |
| **SEX_agree** | 0.010 | 0.002 | 0.008 | 0.001 | 0.001 |
| **DB_agree** | 0.000 | 0.000 | 0.000 | 0.000 | 0.001 |
| **MB_agree** | 0.000 | 0.000 | 0.000 | 0.000 | 0.001 |
| **YB_agree** | 0.000 | 0.000 | 0.000 | 0.000 | 0.001 |
| **TEL_agree** | 0.593 | 0.448 | 0.594 | 0.000 | 0.478 |
| **ADR_agree** | 0.561 | 0.023 | 0.558 | 0.499 | 0.304 |
| **CITY_agree** | 0.568 | 0.004 | 0.566 | 0.525 | 0.310 |
| **ST_agree** | 0.566 | 0.002 | 0.563 | 0.524 | 0.306 |
| **ZIP_agree** | 0.570 | 0.000 | 0.567 | 0.527 | 0.313 |
| **EMAIL_agree** | 0.990 | 0.978 | 0.988 | 0.974 | 0.986 |
