# Supplementary material for: The Data-Adaptive Fellegi-Sunter Model for Probabilistic Record Linkage: Algorithm Development and Validation for Incorporating Missing Data and Field Selection
Source: J Med Internet Res. 2022 Sep 29;24(9):e33775. doi: 10.2196/33775 (PMC9562057; doi:10.2196/33775)
Supplement: Multimedia Appendix 2 [file jmir_v24i9e33775_app2.docx]

**Multimedia Appendix 2**

Table S2 Proportion of missing values by field in the SSA use case. For each blocking scheme (column) the ***unshaded*** fields are used for matching in the final FS model for that block in the data-driven approach.

| **Matching field** | **Blocking scheme** | | | | |
| --- | --- | --- | --- | --- | --- |
|  | **fn-ln-db-mb-yb** | **fn-ln-mi-db-mb** | **fn-ln-mi-yb** | **fn-ln-zip** | **ssn** |
| **MRN_agree** | 1.000 | 1.000 | 1.000 | 1.000 | 1.000 |
| **SSN_agree** | 0.286 | 0.221 | 0.263 | 0.133 | 0.000 |
| **LN_agree** | 0.000 | 0.000 | 0.000 | 0.000 | 0.000 |
| **FN_agree** | 0.000 | 0.000 | 0.000 | 0.000 | 0.001 |
| **Nick_agree** | 0.384 | 0.272 | 0.172 | 0.369 | 0.523 |
| **MI_agree** | 0.602 | 0.000 | 0.000 | 0.462 | 0.589 |
| **ETH_agree** | 0.000 | 0.000 | 0.000 | 0.000 | 0.000 |
| **SEX_agree** | 1.000 | 1.000 | 1.000 | 1.000 | 1.000 |
| **DB_agree** | 0.000 | 0.000 | 0.000 | 0.000 | 0.005 |
| **MB_agree** | 0.000 | 0.000 | 0.000 | 0.000 | 0.005 |
| **YB_agree** | 0.000 | 0.000 | 0.000 | 0.000 | 0.005 |
| **TEL_agree** | 1.000 | 1.000 | 1.000 | 1.000 | 1.000 |
| **ADR_agree** | 1.000 | 1.000 | 1.000 | 1.000 | 1.000 |
| **CITY_agree** | 1.000 | 1.000 | 1.000 | 1.000 | 1.000 |
| **ST_agree** | 0.944 | 0.971 | 0.980 | 0.001 | 0.946 |
| **ZIP_agree** | 0.976 | 0.986 | 0.990 | 0.000 | 0.977 |
| **EMAIL_agree** | 1.000 | 1.000 | 1.000 | 1.000 | 1.000 |
