# Supplementary material for: The Data-Adaptive Fellegi-Sunter Model for Probabilistic Record Linkage: Algorithm Development and Validation for Incorporating Missing Data and Field Selection
Source: J Med Internet Res. 2022 Sep 29;24(9):e33775. doi: 10.2196/33775 (PMC9562057; doi:10.2196/33775)
Supplement: Multimedia Appendix 3 [file jmir_v24i9e33775_app3.docx]

**Multimedia Appendix 3**

Table S3 Proportion of missing values by field in the NBS use case. For each blocking scheme (column) the ***unshaded*** fields are used for matching in the final FS model for that block in the data-driven approach.

| **Matching field** | **Blocking scheme** | | | | |
| --- | --- | --- | --- | --- | --- |
|  | **ln-fn** | **mb-db-zip** | **mrn** | **nk_ln-nk_fn** | **tel** |
| **MRN_agree** | 0.000 | 0.000 | 0.000 | 0.000 | 0.000 |
| **SSN_agree** | 0.981 | 0.988 | 0.981 | 0.980 | 0.980 |
| **LN_agree** | 0.000 | 0.001 | 0.001 | 0.005 | 0.001 |
| **FN_agree** | 0.000 | 0.001 | 0.001 | 0.005 | 0.001 |
| **Nick_agree** | 0.847 | 0.952 | 0.920 | 0.944 | 0.925 |
| **MI_agree** | 0.509 | 0.734 | 0.649 | 0.651 | 0.682 |
| **ETH_agree** | 0.000 | 0.000 | 0.000 | 0.000 | 0.000 |
| **SEX_agree** | 0.003 | 0.009 | 0.012 | 0.026 | 0.009 |
| **DB_agree** | 0.000 | 0.000 | 0.000 | 0.000 | 0.000 |
| **MB_agree** | 0.000 | 0.000 | 0.000 | 0.000 | 0.000 |
| **YB_agree** | 0.000 | 0.000 | 0.000 | 0.000 | 0.000 |
| **TEL_agree** | 0.531 | 0.415 | 0.410 | 0.170 | 0.000 |
| **ADR_agree** | 0.132 | 0.000 | 0.100 | 0.014 | 0.005 |
| **CITY_agree** | 0.133 | 0.000 | 0.101 | 0.014 | 0.005 |
| **ST_agree** | 0.130 | 0.004 | 0.101 | 0.026 | 0.005 |
| **ZIP_agree** | 0.134 | 0.000 | 0.116 | 0.014 | 0.010 |
| **NK_LN_agree** | 0.830 | 0.753 | 0.760 | 0.000 | 0.653 |
| **NK_FN_agree** | 0.831 | 0.754 | 0.761 | 0.000 | 0.654 |
| **DR_LN_agree** | 0.376 | 0.341 | 0.287 | 0.207 | 0.121 |
| **DR_FN_agree** | 0.434 | 0.377 | 0.330 | 0.258 | 0.140 |
| **EMAIL_agree** | 1.000 | 1.000 | 1.000 | 1.000 | 1.000 |
