# Supplementary material for: The Data-Adaptive Fellegi-Sunter Model for Probabilistic Record Linkage: Algorithm Development and Validation for Incorporating Missing Data and Field Selection
Source: J Med Internet Res. 2022 Sep 29;24(9):e33775. doi: 10.2196/33775 (PMC9562057; doi:10.2196/33775)
Supplement: Multimedia Appendix 4 [file jmir_v24i9e33775_app4.docx]

**Multimedia Appendix 4**

Table S4 Proportion of missing values by field in the MCHD use case. For each blocking scheme (column) the ***unshaded*** fields are used for matching in the final FS model for that block in the data-driven approach.

| **Matching field** | **Blocking scheme** | | | | |
| --- | --- | --- | --- | --- | --- |
|  | **db-ln-mb-yb** | **db-mb-yb-zip** | **fn-ln-yb** | **ssn** | **tel** |
| **MRN_agree** | 1.000 | 1.000 | 1.000 | 1.000 | 1.000 |
| **SSN_agree** | 0.735 | 0.713 | 0.724 | 0.000 | 0.623 |
| **LN_agree** | 0.000 | 0.000 | 0.000 | 0.000 | 0.000 |
| **FN_agree** | 0.000 | 0.000 | 0.000 | 0.000 | 0.017 |
| **Nick_agree** | 0.706 | 0.845 | 0.609 | 0.647 | 0.760 |
| **MI_agree** | 0.605 | 0.668 | 0.588 | 0.470 | 0.569 |
| **ETH_agree** | 0.000 | 0.000 | 0.000 | 0.000 | 0.000 |
| **SEX_agree** | 0.025 | 0.008 | 0.024 | 0.002 | 0.007 |
| **DB_agree** | 0.000 | 0.000 | 0.000 | 0.000 | 0.000 |
| **MB_agree** | 0.000 | 0.000 | 0.000 | 0.000 | 0.000 |
| **YB_agree** | 0.000 | 0.000 | 0.000 | 0.000 | 0.000 |
| **TEL_agree** | 0.516 | 0.423 | 0.519 | 0.465 | 0.000 |
| **ADR_agree** | 0.347 | 0.023 | 0.350 | 0.164 | 0.005 |
| **CITY_agree** | 0.343 | 0.002 | 0.349 | 0.156 | 0.013 |
| **ST_agree** | 0.338 | 0.001 | 0.343 | 0.151 | 0.012 |
| **ZIP_agree** | 0.349 | 0.000 | 0.354 | 0.164 | 0.027 |
| **EMAIL_agree** | 0.993 | 0.992 | 0.993 | 0.990 | 1.000 |
