# Supplementary material for: The Data-Adaptive Fellegi-Sunter Model for Probabilistic Record Linkage: Algorithm Development and Validation for Incorporating Missing Data and Field Selection
Source: J Med Internet Res. 2022 Sep 29;24(9):e33775. doi: 10.2196/33775 (PMC9562057; doi:10.2196/33775)
Supplement: Multimedia Appendix 6 [file jmir_v24i9e33775_app6.docx]

**Multimedia Appendix 6**

Table S6 Matching results of the MCHD use case evaluated on a set of 15,500 randomly selected and manually reviewed record pairs. The first two rows are the overall results combined from all blocks on the manually reviewed sample, with the first row for MAD (missing as disagreement) and the second row for MAR (missing at random). Every subsequent two rows pertain to a specific block, with the first containing the results of MAD and the 2^nd^ row the results of MAR. Columns N, SEN, SPE, PPV, NPV and F1 are the total number of manually reviewed record pairs, sensitivity, specificity, positive predictive value, negative predictive value and F-score.

|  |  |  | Expert-Specified Fields | | | | | Data-Driven Fields | | | | |
| --- | --- | --- | --- | --- | --- | --- | --- | --- | --- | --- | --- | --- |
| Overall |  | N | SEN | SPE | PPV | NPV | F1 | SEN | SPE | PPV | NPV | F1 |
|  | **MAD** | 15500 | 0.944 | 0.989 | 0.982 | 0.966 | **0.963** | 0.635 | 0.970 | 0.929 | 0.811 | **0.754** |
|  | **MAR** | 15500 | 0.946 | 0.988 | 0.980 | 0.967 | **0.963** | 0.954 | 0.988 | 0.979 | 0.972 | **0.967** |
| Per block | **db-ln-mb-yb** | 6704 | 0.313 | 0.971 | 0.979 | 0.242 | 0.475 | 0.552 | 0.814 | 0.930 | 0.291 | 0.693 |
|  | **db-ln-mb-yb_mar** | 6704 | 0.953 | 0.964 | 0.992 | 0.823 | 0.972 | 0.963 | 0.955 | 0.990 | 0.854 | 0.976 |
|  | **db-mb-yb-zip** | 9208 | 0.943 | 0.992 | 0.970 | 0.985 | 0.957 | 0.933 | 0.993 | 0.973 | 0.982 | 0.953 |
|  | **db-mb-yb-zip_mar** | 9208 | 0.990 | 0.990 | 0.964 | 0.997 | 0.977 | 0.966 | 0.992 | 0.969 | 0.991 | 0.967 |
|  | **fn-ln-yb** | 6118 | 0.991 | 0.986 | 0.997 | 0.954 | 0.994 | 0.542 | 0.981 | 0.993 | 0.298 | 0.701 |
|  | **fn-ln-yb_mar** | 6118 | 0.991 | 0.974 | 0.995 | 0.957 | 0.993 | 0.994 | 0.976 | 0.995 | 0.969 | 0.995 |
|  | **ssn** | 1580 | 0.998 | 0.816 | 0.994 | 0.930 | 0.996 | 0.999 | 0.816 | 0.994 | 0.952 | 0.996 |
|  | **ssn_mar** | 1580 | 0.998 | 0.816 | 0.994 | 0.930 | 0.996 | 0.999 | 0.837 | 0.995 | 0.953 | 0.997 |
|  | **tel** | 73 | 1.000 | 0.980 | 0.960 | 1.000 | 0.980 | 1.000 | 1.000 | 1.000 | 1.000 | 1.000 |
|  | **tel_mar** | 73 | 1.000 | 0.980 | 0.960 | 1.000 | 0.980 | 1.000 | 1.000 | 1.000 | 1.000 | 1.000 |
