# Supplementary material for: The Data-Adaptive Fellegi-Sunter Model for Probabilistic Record Linkage: Algorithm Development and Validation for Incorporating Missing Data and Field Selection
Source: J Med Internet Res. 2022 Sep 29;24(9):e33775. doi: 10.2196/33775 (PMC9562057; doi:10.2196/33775)
Supplement: Multimedia Appendix 8 [file jmir_v24i9e33775_app8.docx]

**Multimedia Appendix 8**

Table S8 Data quality of fields of last name and first name in the DOB-ZIP block of the INPC and NBS use cases

|  | **Probability of**  **agreement** | **INPC** | **NBS** |
| --- | --- | --- | --- |
| **Last name** | **matches** | 0.9445 | 0.8900 |
|  | **non-matches** | 0.0045 | 0.0270 |
| **First name** | **matches** | 0.9436 | 0.6699 |
|  | **non-matches** | 0.0040 | 0.0345 |
